# The influence of the aortic valve angle on the hemodynamic features of the thoracic aorta

**DOI:** 10.1038/srep32316

**Published:** 2016-08-26

**Authors:** Hojin Ha, Guk Bae Kim, Jihoon Kweon, Sang Joon Lee, Young-Hak Kim, Namkug Kim, Dong Hyun Yang

**Affiliations:** 1 POSTECH Biotech Center, Pohang University of Science and Technology, San 31, Hyoja-dong, Pohang 790-784, South Korea; 2 Asan Institute of Life Science, Asan Medical Center, University of Ulsan College of Medicine, 388-1 Poongnap-dong, Songpa-gu, Seoul 138-736, South Korea; 3 Department of Cardiology, University of Ulsan College of Medicine, Asan Medical Center, 388-1 Poongnap-dong, Songpa-gu, Seoul 138-736, South Korea; 4 Department of Mechanical Engineering, Pohang University of Science and Technology (POSTECH), San 31, Hyoja-dong, Pohang 790-784, South Korea; 5 Department of Convergence Medicine, University of Ulsan College of Medicine, Asan Medical Center, 388-1 Poongnap-dong, Songpa-gu, Seoul 138-736, South Korea; 6 Department of Radiology, University of Ulsan College of Medicine, Asan Medical Center, 388-1 Poongnap-dong, Songpa-gu, Seoul 138-736, South Korea

## Abstract

Since the first observation of a helical flow pattern in aortic blood flow, the existence of helical blood flow has been found to be associated with various pathological conditions such as bicuspid aortic valve, aortic stenosis, and aortic dilatation. However, an understanding of the development of helical blood flow and its clinical implications are still lacking. In our present study, we hypothesized that the direction and angle of aortic inflow can influence helical flow patterns and related hemodynamic features in the thoracic aorta. Therefore, we investigated the hemodynamic features in the thoracic aorta and various aortic inflow angles using patient-specific vascular phantoms that were generated using a 3D printer and time-resolved, 3D, phase-contrast magnetic resonance imaging (PC-MRI). The results show that the rotational direction and strength of helical blood flow in the thoracic aorta largely vary according to the inflow direction of the aorta, and a higher helical velocity results in higher wall shear stress distributions. In addition, right-handed rotational flow conditions with higher rotational velocities imply a larger total kinetic energy than left-handed rotational flow conditions with lower rotational velocities.

Understanding the hemodynamic features of blood flow in a blood vessel is important because they are closely related to the development of cardiovascular diseases^1^. Pathological hemodynamic conditions, such as abnormal wall shear stress (WSS) or locally stagnant blood flow, can cause vascular diseases by initiating abnormal morphological and functional changes in the endothelial cell layer^2^,^3^. Various clinical studies also support the association between abnormal hemodynamic features and various vascular diseases, such as abnormally high WSS in the ascending aorta and helical aortic flow in patients with aortic valve stenosis (e.g., bicuspid aortic valve)^4^,^5^,^6^.

Various studies have found that the existence of helical blood flow is associated with various pathological conditions, such as bicuspid aortic valve (BAV), aortic stenosis, aortic dilatation, and tortuosity of the aorta^7^,^8^,^9^. In particular, the patient with aortic stenosis frequently develops helical blood flow compared to the normal subject (Fig. 1, also refs 7, 8, 9). Some studies proposed using the existence and intensity of helical flow as a fluid-dynamic risk factor that is indicative of vascular disease^10^,^11^,^12^. However, knowledge of the development of helical blood flow and its biological and clinical implications are still not completely understood. Therefore, understanding the causes and influences of helical blood flow is important for the further development of risk prediction models and potential interventions in the future.

Recently, time-resolved, 3D, phase-contrast (PC) magnetic resonance imaging (MRI)—which is also called 4D PC-MRI–was used to investigate spatial and temporal variations in the hemodynamic features of blood flow^13^,^14^. The streamlines and pathlines calculated from the time-resolved 3D velocity distributions have the potential to visualize complex blood flow structures, including helical flow patterns. In addition, recent advances in 3D modeling and printing techniques facilitate the fabrication of patient-specific flow phantoms. While *in vivo* studies do not predict hemodynamic changes due to various vascular modifications, vascular flow phantoms facilitate in-depth fluid-dynamic experiments with various modifications for vascular geometries^15^,^16^.

Previous studies show that patients with aortic stenosis frequently demonstrate various types of helical blood flow in the thoracic aorta^5^, and we hypothesized that the direction and angle of aortic valve flow can influence the flow patterns in the thoracic aorta. Therefore, in the present study, our aim was to investigate the influence of the aortic flow angle on the hemodynamic features in the thoracic aorta using 3D-printed vascular flow phantoms and 4D PC-MRI measurements and quantifications. In particular, the development of helical blood flow, which depends on the direction of aortic valve flow, and the associations between helical blood flow and various fluid-dynamic indices such as the streamline, WSS, and turbulence kinetic energy (TKE), were investigated to understand if helical flow characteristics are appropriate fluid-dynamic risk predictors of vascular disease.

## Results

### Development of helical blood flow

**Figure 2:**
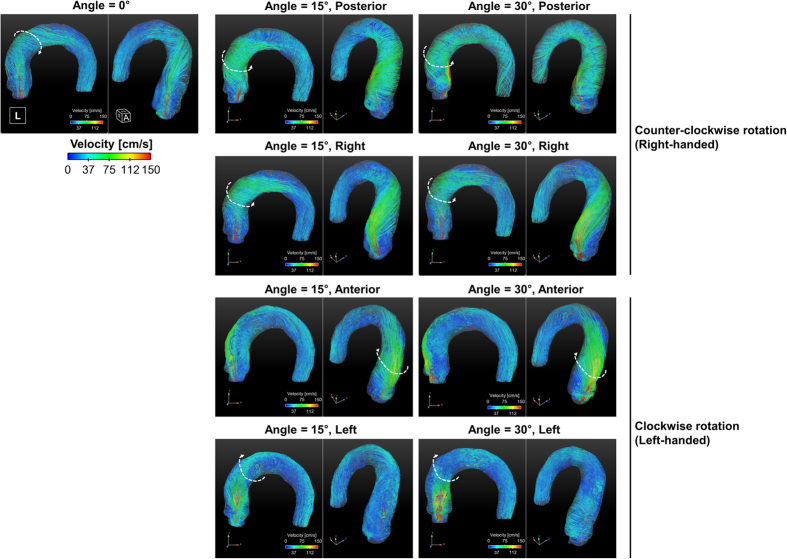
Streamlined visualization of aortic flows with various directional aortic valves. Note that the helical flow directions varied depending on the aortic valve flows. The straight, posterior, and right directions of the aortic valve flows resulted in right-handed rotations in the aorta, while the anterior and left directions of the aortic valve flows resulted in left-handed rotations in the aorta. A indicates anterior; L, left; R, right; H, head.

**Figure 3:**
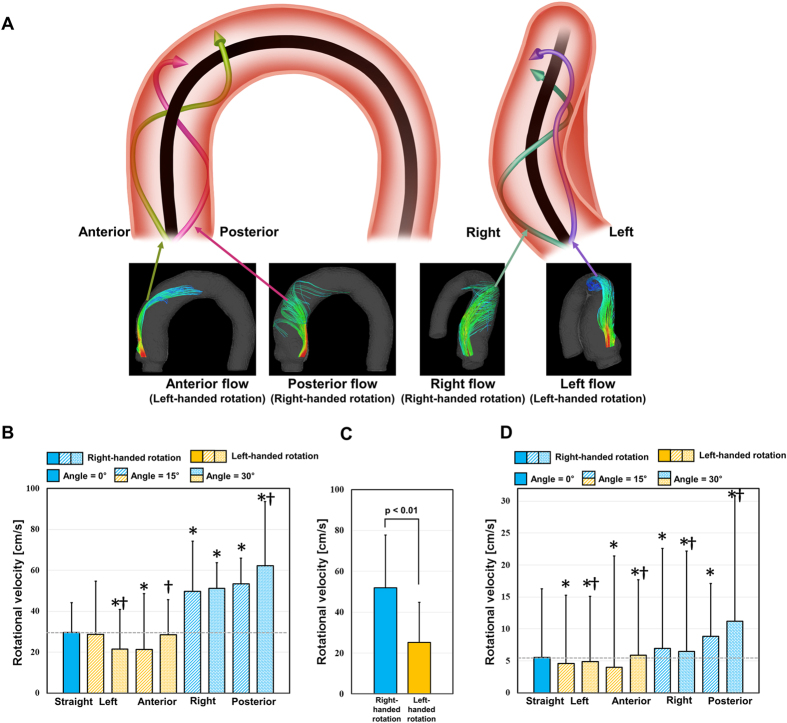
Helical rotations of various aortic valve flows. (**A**) Development of rotational flows in the ascending aorta, (**B**) rotational velocity in the ascending aorta, (**C**) comparison of rotational velocity in the ascending aorta depending on the helical rotation directions, and (**D**) average rotational velocity of the whole aortic flow. Note that the black solid line in (**A**) is the centerline axis of the thoracic aorta. *Statistical difference (*p* < 0.01, Bonferroni corrected) in comparison with the rotational velocity of straight aortic valve flow. ^†^Statistical difference (*p* < 0.01, Bonferroni corrected) in comparison with the rotational velocity in the same aortic flow direction, but at 15°. The error bar indicates the mean + SD. Only positive errors are shown for clarity.

Flow through the thoracic aorta with a straight aortic inflow (angle = 0°) has a slight helical, counter-clockwise, right-handed rotation (Fig. 2). The rotational direction of helical blood flow varies depending on the direction of aortic valve flow. Aortic valve flows toward the posterior and right directions induce right-handed rotation, while flows toward the anterior and left directions induce left-handed (i.e., clockwise) rotation (see Fig. 3A). The direction of helical blood flow is more dependent on the direction of the aortic valve flow than the angle of the flow; therefore, an increase in the aortic flow angle from 15° to 30° does not change the direction of helical blood flow.

### Helical flow direction according to the inflow angle

The helical component of flow mostly develops at the ascending aorta when the aortic jet flow impinges on the ascending aorta (Fig. 3A). Redirecting flow at the ascending aorta determines if the flow has a right-handed or left-handed rotation. In our present study, 4D PC-MRI showed that the anterior- and posterior-directional aortic flows naturally generate left- and right-handed rotations around the centerline axis of the thoracic vessel, respectively, as they are combined with the left-to-right directional velocity components. On the other hand, right- and left-directional aortic flows develop helical components due to the curvature of the thoracic aorta at the ascending aorta. Due to the curvature of the thoracic aorta, the right- and left-directional aortic flows travel toward the outer curvature of the aorta and develop right- and left-handed rotation, respectively.

### Rotational velocity according to the helical flow direction

In comparison with the rotational velocity at the ascending aorta with straight aortic flow, flows with left-handed rotation demonstrated a significantly lower rotational velocity at the ascending aorta, except left-directional flow at 15° (*p* < 0.01, Bonferroni corrected; Fig. 3B). In contrast, flows with right-handed rotation demonstrated a significantly higher rotational velocity at the ascending aorta (*p* < 0.01, Bonferroni corrected; Fig. 3B). The increase in the aortic flow angle from 15° to 30° increased the rotational velocity at the ascending aorta, except aortic flow directed toward the left (*p* > 0.01, Bonferroni corrected). Consequently, the flow group with right-handed rotation (right and posterior aortic valve flows at 15° and 30°, respectively) demonstrated a significantly higher rotational velocity than the flow groups with left-handed rotation (left and anterior aortic valve flows at 15° and 30°, respectively) (*p* < 0.01; Fig. 3C).

Comparing the mean rotational velocity in the overall thoracic aorta with the straight aortic flow, the flows with left-handed rotation demonstrated significantly lower rotational velocities at the ascending aorta, except anterior directional flow at 30° (*p* < 0.01, Bonferroni corrected) (Fig. 3D). In contrast, flows with right-handed rotation demonstrated significantly higher rotational velocities in the thoracic aorta (*p* < 0.01, Bonferroni corrected). Increasing of the aortic flow angle from 15° to 30° increased the overall rotational velocity in the thoracic aorta, except right directional flow at 30°.

### Vortical structure in aortic flow

**Figure 4:**
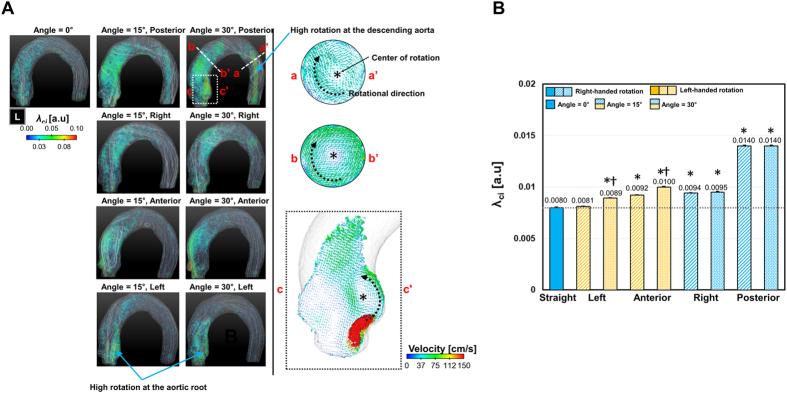
Comparison of λ_ci_ for aortic flows with various aortic valve directions. (**A**) Volumetric visualization of λ_ci_ and representative cross-sectional velocity fields at high λ_ci_ regions. Note that λ_ci_ indicates the local intensity of rotational flow. (**B**) Plot of the average λ_ci_ in the thoracic aorta. *Indicates statistical differences (*p* < 0.01, Bonferroni corrected) in comparison with the rotational velocity of the straight aortic valve flow. ^†^Indicates statistical differences (*p* < 0.01, Bonferroni corrected) in comparison with the rotational velocity in the same aortic flow direction, but at 15°. The error bar indicates the mean ± SE. L indicates the left direction.

The vortex identification method, which is based on critical point analysis of the local velocity’s gradient tensor and its corresponding eigenvalue, visualized the locations and the strengths of the local vortical flows in the thoracic aorta (Fig. 4A). While straight-directional aortic flow induced only minor vortices around the aortic root and ascending aorta, aortic flows with the posterior-directional aortic valve flows demonstrated λ_ci_ > 0.05 at the aortic root, ascending aorta, and even the descending aorta. Those regions with non-zero λ_ci_ values were confirmed to have a coherent rotational velocity field at the aortic root (c-c’ in Fig. 4A), ascending aorta (b-b’ in Fig. 4A), and descending aorta (a-a’ in Fig. 4A). On the other hand, the right-, anterior-, and left-directional aortic valve flows induced vortical flow structures, mostly around the aortic root or ascending aorta. Resultantly, in comparison with straight aortic valve flow, aortic valve flows in 4 different directions (left, right, anterior, and posterior) and 2 different angles (15° and 30°) demonstrated significant increases in the mean λ_ci_ value for aortic flow, except the left at 15° (*p* < 0.01, Bonferroni corrected; Fig. 4B). In addition, aortic valve flows at 30° demonstrated significantly higher λ_ci_ values in comparison with those at 15°, except under posterior- and right-directional aortic flow conditions (*p* < 0.01, Bonferroni corrected; Fig. 4B).

### WSS distributions in the aorta

**Figure 5:**
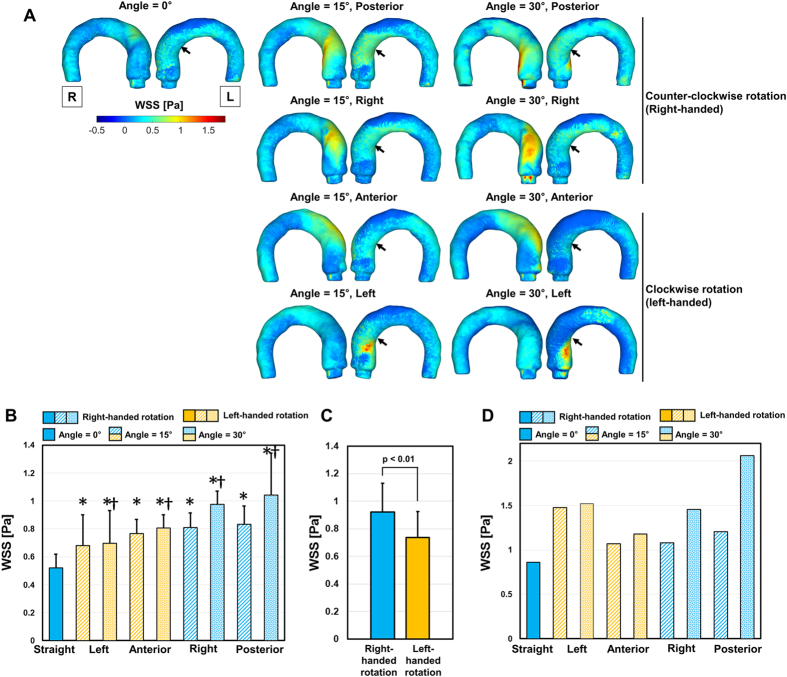
Effect of the aortic flow direction and angle on WSS in the thoracic aorta. (**A**) Colormap of WSS, (**B**) comparison with the top 5% percentile of WSS, (**C**) comparison with the top 5% percentile of WSS depending on the rotational direction, and (**D**) comparison of the maximum WSS at the thoracic aorta. The black arrows in (**A**) indicate the region of WSS in the ascending aorta. *Statistical difference (*p* < 0.01, Bonferroni corrected) in comparison with WSS at straight aortic valve flow. ^†^Statistical difference (*p* < 0.01, Bonferroni corrected) in comparison with WSS in the same aortic flow direction, but at 15°. The error bar indicates mean + SD. Only the positive error is shown for clarity. L and R indicate left and right, respectively.

The WSS distribution in the thoracic aorta was highly dependent on the directions of the aortic valve flows. Straight aortic flow induced WSS < 1.0 Pa because the high-velocity jet flow dissipates due to its long travel length until impinging on the ascending aorta. On the contrary, aortic valve flows at 15° and 30° demonstrated less velocity dissipation due to a shorter travel length until it impinged on the vessel, so these flows induced local maximum WSS values > 1.0 Pa (Fig. 5A,D). In comparison to WSS measured at anterior- and left-directional aortic valve flows, WSS at posterior- and right-directional aortic valve flows demonstrated relatively high WSS values at the ascending aorta (as indicated by the arrows in Fig. 5A), mostly due to the higher rotational velocity in the corresponding region. The quantitative analysis of WSS also showed that aortic valve flows at 15° and 30° significantly increased the top 5% percentile of WSS (*p* < 0.01) in comparison with WSS induced by straight aortic valve flow. In addition, aortic valve flows at 30° demonstrated significantly higher WSS in comparison with those at 15° (*p* < 0.01, Bonferroni corrected; Fig. 5B). Due to the differences in the rotational velocity, the overall top 5% of WSS in the flow group with right-handed rotation was higher than that of the flow group with left-handed rotation (Fig. 5C).

**Figure 6:**
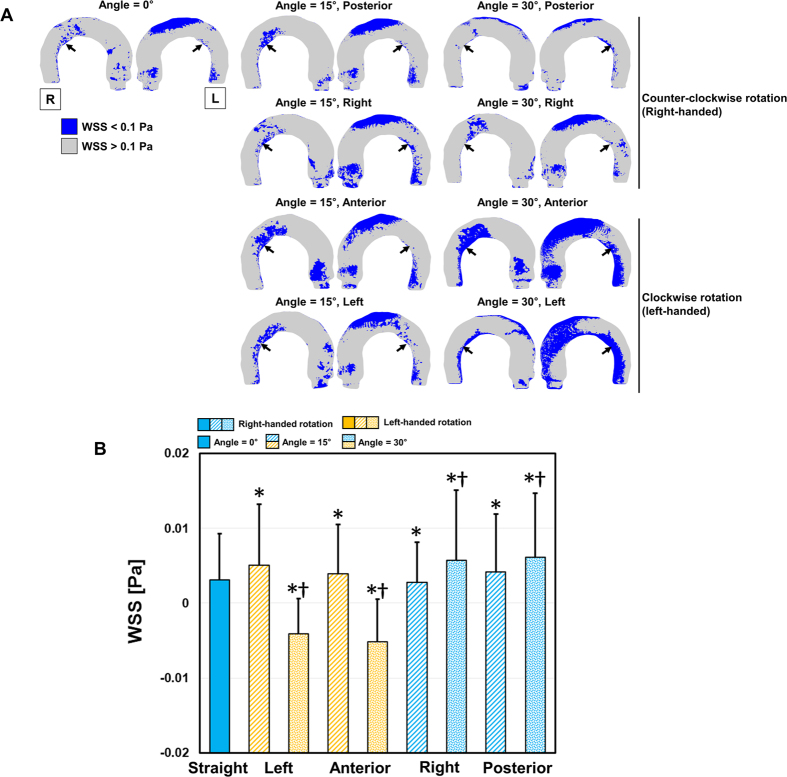
Low WSS distribution in the thoracic aorta. (**A**) Colormap of WSS where WSS is <0.1 Pa, (**B**) comparison with the bottom 5% percentile of WSS. The black arrows in (**A**) indicate regions of WSS with <0.1 Pa in the descending aorta. *Statistical difference (*p* < 0.01, Bonferroni corrected) in comparison with WSS at straight aortic valve flow. ^†^Statistical difference (*p* < 0.01, Bonferroni corrected) in comparison with WSS at the same aortic flow direction, but at 15°. The error bar indicates the mean + SD. Only the positive error is shown for clarity. L and R indicate left and right, respectively.

Low WSS distributions were also influenced by the helical flow patterns of different aortic valve flows. Straight aortic valve flow induced a relatively low WSS distribution (<0.1 Pa) at the aortic root and the outer and inner curvature regions of the thoracic aorta (Fig. 6A). In the present study, the increase in the aortic valve angle from 15° to 30° in the posterior- and right-directional flows reduced those low WSS distributions, mostly due to the increase in the helical flow components, while the increase in the aortic valve angle from 15° to 30° in the anterior- and left-directional flows increased in low-WSS regions (Fig. 6A,B).

### Elevation of local pressure by impinging flow

**Figure 7:**
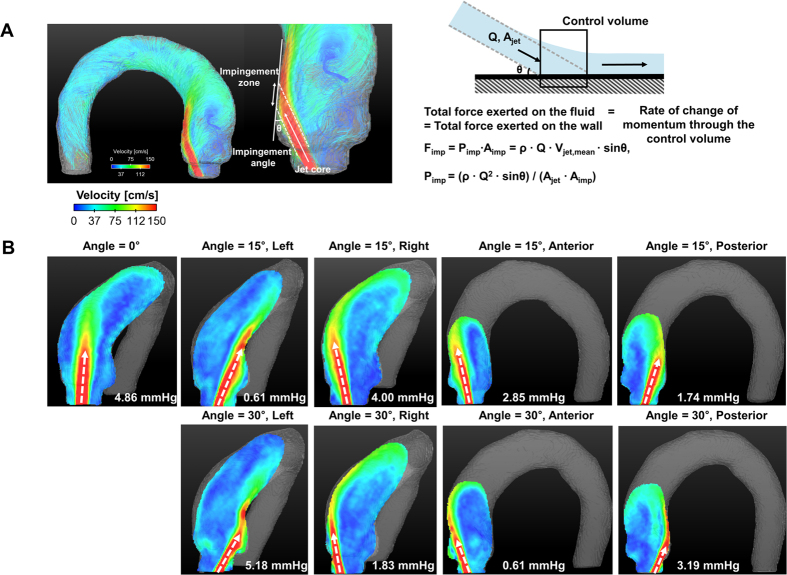
Effect of the aortic valve direction and angle on the impinging pressure at the aorta. (**A**) Principle and basic parameters for estimating impinging pressure, and (**B**) flow impinging patterns and corresponding impinging parameters at various aortic valve flows. The white dashed arrows in (**B**) indicate the directions of the aortic valve flows.

Impinging pressure was highly dependent on the contact angle between the flow and the aortic vessel, rather than the helical nature of overall flow (Fig. 7A). Therefore, the direction and the degree of the aortic valve flow alone did not result in the general trends in impinging pressure (P_imp_) variations. Straight- and left-directional aortic flow at 30° demonstrated a relatively high P_imp_ value (>4.5 mmHg) because the contact angle between the vessel surface and the flows were relatively higher (46.13° ± 1.41° and 38.63° ± 1.20° for straight and left-directional aortic valve flows at 30°, respectively) (Table 1). On the contrary, aortic flows with left- and posterior-directional flows at 15° and right- and anterior-directional flows at 30° resulted in P_imp_ values < 2.0 mmHg because the directions of the jet flows were well aligned with the contact surface of the vascular wall (11.96° ± 1.88° and 20.43° ± 0.76° for the left- and posterior-directional aortic flows at 15°, and 21.18° ± 1.18° and 12.02° ± 2.17° for the right- and anterior-directional aortic flows at 30°, respectively) (Table 1).

### Turbulence kinetic energy

**Figure 8:**
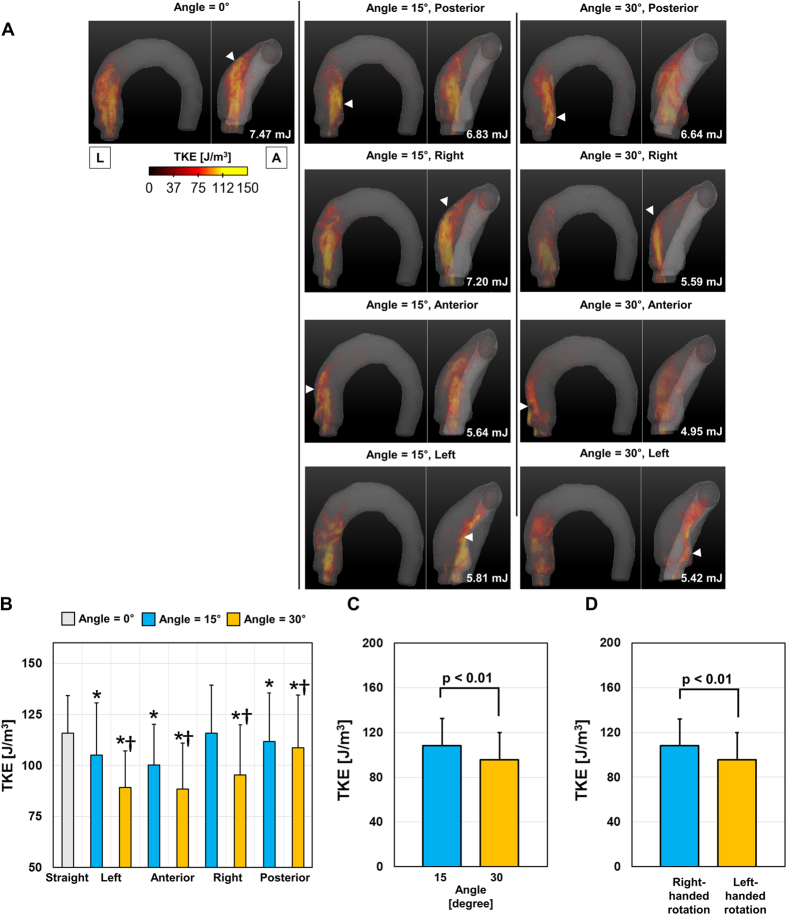
Effect of the aortic valve direction and angle on TKE distribution. (**A**) Volume-rendering of TKE in the thoracic aorta, (**B**) comparison with the top 5% percentile of TKE, (**C**) effect of the aortic valve angle, and (**D**) effect of helical rotation. *Statistical difference (*p* < 0.01, Bonferroni corrected) in comparison with TKE at the straight aortic valve flow. ^†^Statistical difference (*p* < 0.01, Bonferroni corrected) in comparison with TKE at the same aortic flow direction, but at 15°. The error bar indicates the mean + SD. Only the positive error is shown for clarity.

Aortic flow results in a high turbulence kinetic energy (TKE) distribution, where the jet flow from the aortic valve interfaces with the surrounding fluid because the interface is where the unstable turbulent vortices develop. Therefore, straight aortic valve flow generates the highest TKE distribution because the straight jet flow has a long length to travel until it impinges on the ascending aorta and a large interfacial region between the jet flow and surrounding flow (Fig. 8A). On the other hand, the aortic flows with angular deflections (left, right, posterior, and anterior directions both 15° and 30°) demonstrated a lower TKE distribution, as the TKE of the flow rapidly dissipates after the flow impinges on the vascular wall (Fig. 8A). Therefore, the top 5% percentile of TKE was the highest with straight aortic valve flow. In addition, an aortic valve angle of 30° significantly reduced TKE in comparison with TKE at 15° (Fig. 8B,C). Overall, the flow group with right-handed rotation demonstrated a significantly larger TKE in comparison with the group with left-handed rotation (*p* < 0.01; Fig. 8D).

**Figure 9:**
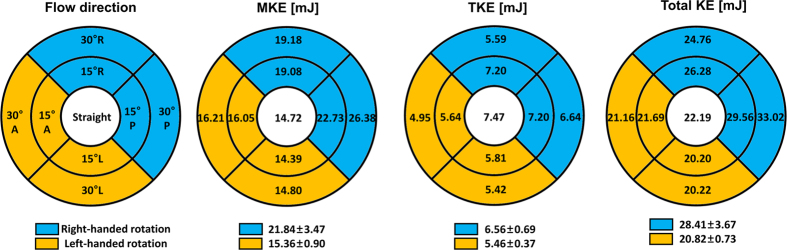
Distributions of MKE, TKE, and total KE at various angles and the directions of the aortic valve flows. L and A indicate left and anterior, respectively.

Mean kinetic energy (MKE), TKE, and the total kinetic energy (KE) were compared using a circular map (Fig. 9). The maps show that right-handed rotation tends to induce higher MKE and TKE values in comparison with left-handed rotation (MKE = 21.83 ± 3.47 mJ and 15.36 ± 0.90 mJ; TKE = 6.56 ± 0.67 mJ and 5.46 ± 0.37 mJ for right- and left-handed rotations, respectively). As a result, right-handed rotation demonstrated a higher total KE in comparison with left-handed rotation (28.41 ± 3.67 mJ and 20.82 ± 0.73 mJ for right- and left-handed rotations, respectively).

## Discussion

The major findings of our present study included the following: (a) the rotational direction and strength (λ_ci_) of helical blood flow in the thoracic aorta varies according to the direction of the aortic valve flow; (b) aortic flows with higher helical velocity components have higher WSS distributions, even when the blood flow rate and diameter of the aortic valve are controlled; and (c) right-handed rotational flow conditions with higher rotational velocities have larger TKE, MKE, and total KE values than left-handed rotational flow conditions with lower rotational velocities.

Several groups have reported that patients with bicuspid aortic valve (BAV) disease have abnormally high WSS distributions at the ascending aorta because of malformations of the aortic valve, which are frequently accompanied by aortic valve stenosis and high WSS at the ascending aorta. Therefore, abnormal WSS is considered a major contributor to aortic dilation in BAV^6^,^17^. The severity of aortic regurgitation is also correlated with the degree of aortic root dilatation because regurgitation induces higher stroke volumes and leads to higher aortic jet flows and WSS in the ascending aorta^18^,^19^. Considering the fact that the risk factors based on the geometric severity of stenosis alone are not successful indicators of aortic dilatation, and the correlation between stenosis severity and aortic dilatation is conflicted^18^,^20^,^21^, the quantification of hemodynamic features is rather promising for predicting and diagnosing vascular diseases.

The origin and possible influences of helical blood flow are still questionable. Previously, Liu *et al*.^22^ discussed that existing helical flow may also play positive physiological roles in enhancing blood flow transport, suppressing disturbed blood flow, preventing the accumulation of atherogenic low density lipoproteins on the luminal surfaces of arteries, enhancing oxygen transport from the blood to the arterial wall and reducing the adhesion of blood cells on the arterial surface^22^. However, helical blood flow in the thoracic aorta has been also observed in patients with aortic stenosis, BAV, and aortic dilatation^5^,^23^,^24^. Therefore, the role of the helical blood flow seems to be different with the pathological and physiological conditions. The present study also found that the rotational direction and strength (λ_ci_) of helical blood flow in the thoracic aorta varies according to the direction of aortic valve flow. In particular, aortic valve flow toward the posterior and right directions tended to generate right-handed rotations, while the left and anterior directions resulted in left-handed rotations (Fig. 2). In addition, higher helical blood flow resulted in a higher WSS distribution in the thoracic aorta (Fig. 5). Since the posterior and right directional aortic flows induced right-handed helical flow with a larger rotational flow velocity, they also demonstrated larger WSS in comparison to the anterior- and left-directional aortic flows with left-handed helical flow. These results agree well with previous clinical observations in BAV patients, which show that the posterior right-directional and left-directional aortic valve flows induced by different fusion types of BAV result in the right- and left-handed rotations in the thoracic aorta, respectively (see Fig. 1 in ref. 5). In addition, it was also found that BAV predominantly demonstrated abnormal right-handed helical flow in the ascending aorta and larger ascending aortas, systole flow angle, and WSS in comparison with the normal group^5^. According to our present results, the direction and angle of the aortic valve flow due to the formation of BAV can be one of the most important factors that influences the direction and strength of the helical flow, magnitude of WSS, and consequently aortic dilatation.

The direction of aortic valve flow in BAV can depend on the fusion type of the aortic valves. A previous BAV study with PC-MRI showed that right-left (RL) aortic leaflet fusion dominantly generated rightward blood flow, while right-noncoronary (RN) aortic leaflet fusion induced leftward blood flow^25^. According to our current findings, RL-BAV with rightward blood flow should generate right-handed rotational blood flow, and clinical observations obtained using 4D PC-MRI also support the notion that RL-BAV predominantly develops right-handed rotational blood flow^5^. Accordingly, RN-BAV with leftward blood flow is expected to generate left-handed rotational flows; however, clinical observations in patients with RN-BAV and left-handed rotational blood flows are still lacking^5^,^25^. However, it is also noteworthy that the aortic flow direction can sensitively vary according to aortic valve stenosis or the motion of the leaflets, and determination of the aortic flow direction according to the fusion type of BAV alone might be misleading.

Since the helicity of flow is known to arrest energy decay by inhibiting energy flux from larger to smaller scales^26^, helical blood flow avoids the excessive dissipation of energy by limiting flow instability in the arteries^27^,^28^. Consequently, helical flow with rotational stability is suspected to reduce TKE by its rotational stability^29^. In the present study, while helical blood flow was assumed to reduce total TKE in the thoracic aorta, we did not find any evidence of helical blood flows for the fluid-dynamic stabilities. TKE at the thoracic aorta was predominantly dependent on the length of travel of the aortic jet flow until it impinged on the ascending aorta. TKE was the highest with straight aortic flow, and the increase in the aortic flow angle from 15° to 30° was found to reduce the TKE distribution because it shortened the distance between the aortic valve and the flow-impinging location of the vessel. In addition, it was found that right-handed helical flows with higher rotational velocities demonstrated larger TKE values in comparison to left-handed helical flows with lower rotational velocities. These results agree well with those of a previous study, which showed that high-intensity helical flow does not change the total integral of the turbulence velocity fluctuations^30^.

By contrast, it was found that right-handed rotational flow with higher rotational velocities demonstrated larger TKE, MKE, and total KE values than left-handed rotational flow with lower rotational velocities (Fig. 9). After neglecting other possible energy losses during energy transfer from the heart to blood flow, this indicates that more energy is required for the heart to make the stationary blood in a diastole phase have the higher total KE in the systole phase. Therefore, the increase in the helical velocity components may imply an additional burden for the left ventricle from the point of view of fluid dynamics. However, the clinical evidence of helical blood flow and its effect on morphological and functional changes in the left ventricle have not been investigated.

Although right-handed helical flows with a high rotational velocity result in the development of pathological hemodynamic features, such as increased overall WSS and total KE, they also provide beneficial effects by suppressing low-WSS regions (Fig. 6). In particular, posterior- and right-directional aortic valve flows, which result in high-intensity right-handed rational flows, also resulted in fewer low-WSS regions (WSS < 0.1 Pa) in comparison with the anterior- and left-directional aortic valve flows, which developed low-intensity left-handed rational flows. While abnormally high WSS is suspected to play a role in aortic dilatation^5^,^6^,^31^, low-WSS regions are also susceptible to atherosclerosis due to edothelia cell (EC) inflammation and the high deposition of low-density lipoproteins (LDLs)^32^,^33^,^34^. Previously, a numerical study showed that helical flow reduced the luminal surface of the LDL concentration in the aortic arch and played a role in suppressing the severe polarization of LDL in the thoracic aorta^33^. The present study also agrees with the beneficial effects of helical blood flow by suppressing pathologically low WSS in the thoracic aorta.

The present study also found that aortic flow induces different amounts of impinging pressure depending on the angle between the aortic jet flow and the contact surface of the vessel. Previously, vascular regions with local flow impingement were highly associated with intimal hyperplasia in the artery, although WSS at the corresponding region was low^35^,^36^,^37^. Therefore, we believe that the impinging pressure should also be estimated as a fluid-dynamic risk factor because this parameter is independent of other previously identified fluid-dynamic parameters, such as TKE and WSS. In the present study, right- and anterior-directional aortic flow at 30° resulted in lower P_imp_ in comparison to 15° because the impinging angle of the aortic jet flow was reduced despite the increase in the angle of aortic valve flow. In addition, straight aortic flow and left-directional flow at 30° induced high P_imp_ (>4.5 mmHg) at the ascending aorta and near aortic root, respectively, but induced relatively low WSS in comparison with other flow conditions. Consequently, integrating these contradictory fluid-dynamic factors may better explain what has not been fully understood with the use of a single WSS parameter^4^.

Our present study findings demonstrate that the fabrication of patient-specific aorta phantoms is effective for investigating hemodynamic changes in the thoracic aorta with various directional aortic valves. The use of 3D-printed vascular flow phantoms facilitated in-depth fluid-dynamic experiments and consequently helps to understand the hemodynamic features of pathological conditions that could be used to provide important information for planning surgeries and interventions in the future^38^. Therefore, the combination of 3D-printed, patient-specific phantoms and fluid-dynamic quantification with 4D PC-MRI will become more important as evidence of its clinical importance grows.

A recent study reported that patients with severe aortic stenosis, who underwent pre-operative cardiac magnetic resonance imaging, were found to have severe angular blood flow from the aortic valve^39^. According to the study, the aortic stenosis patients with TAV and BAV had 29.4 ± 8.3° and 26.9 ± 4.8°, respectively^39^. Therefore, in the present study, the geometry of the aorta from one patient with severe aortic stenosis, who had severe angular aortic blood flow around 30°, was representatively employed, and two aortic flow angles (15° and 30 °) were used to simulate the moderate and severe angular blood flow from the aortic valve, respectively. The present study shows that variation of the aortic flow angle with the development of aortic stenosis can influence WSS distributions, TKE, MKE, and total KE values. In addition, those were also varied depending on the rotational direction of the aortic blood flow, which emphasized the importance of 4D PC-MRI for the patients with aortic stenosis.

Previous studies showed that the patients without aortic stenosis did not have high rotational helical flow, WSS and TKE distribution in the ascending aorta, on the contrary to the patients with the aortic stenosis^6^,^40^,^41^,^42^. In addition, the aortic flow angle was mostly found to be severe when the patients had the aortic stenosis^39^. Since the present study aimed to investigate the influence of the aortic flow angle of the aortic stenosis on the hemodynamic features in the thoracic aorta, the flows in the healthy patients were not covered in the present study.

The present study employed 4D PC-MRI with 3D printing for analyzing the aortic blood flow so that the present findings can be also compared with the *in-vivo* aortic flows from patients with the similar measurement environment. However, it is also noteworthy that other experimental and numerical methodologies can be usefully used for *in-vitro* or theoretical studies such as particle image velocimetry, particle tracking velocimetry or computational fluid-dynamics^30^,^42^,^43^,^44^,^45^. Considering the limited spatial resolution of 4D PC-MRI, those experimental and numerical methods can be also good alternative for the hemodynamic research.

A limitation of our present study was that it investigated steady-flow conditions close to systole blood flow but pulsation effects were not investigated. Consequently, the influence of helical blood flow on hemodynamic factors during the decelerating diastole phase was not investigated. Since the influence of the rotational flow component can be more dominant when axial flow is reduced during diastole, further experiments will be needed to evaluate the influence of pulsatile flow. In addition, the aortic model in the present study was simplified without three branches from aortic arch. Since the present study only aimed to prove the influence of the aortic flow angle on the thoracic aortic flow, those effect from the branches were simplified and considered negligible. However, it is also noteworthy that the influence of the aortic branches on the aortic hemodynamics should be included in the next step of the study.

## Conclusion

The rotational direction and strength (λ_ci_) of helical blood flow in the thoracic aorta varies according to the direction of the aortic valve flow. Aortic flows with higher helical velocity components demonstrate higher WSS distributions, and right-handed rotational flow conditions with higher rotational velocities demonstrate larger TKE, MKE, and total KE values than left-handed rotational flow conditions with lower rotational velocities.

## Materials and Methods

### Preparation of thoracic aorta phantoms

The schematic procedures of the experiments are described in Figure S1 of the Supporting Information. CT images of an 84-year-old man with severe aortic stenosis were used for 3D modelling. CT was performed to preoperatively evaluate the thoracic aorta. Echocardiography revealed severe degenerative aortic stenosis with a markedly elevated trans-valvular pressure gradient (mean = 42 mmHg). Because of the retrospective use of the data included in this study, the institutional review board at our institution did not require written informed consent. CT scans were performed using second-generation, dual-source CT (Somatom Definition Flash; Siemens, Erlangen, Germany) after the intravenous injection of the contrast material. 3D models of the thoracic aorta were processed using in-house software (AView; Department of Convergence Medicine, Asan Medical Center, South Korea). Segmentation of the thoracic aorta was processed using a combination of thresholding and a region-growing algorithm. Then, the left and right subclavian arteries and left common artery were removed from the model using a 3D sculpture algorithm in order to simplify the shape of the thoracic aorta (Figure S1 in the Supplementary information). Finally, the surface of the model was smoothed using the shrink-smoothen and robust-smoothen functions of Meshmixer (Autodesk, San Rafael, CA). At the inlet of the aortic root, the aortic valve with stenosis was simplified as a circular-shaped hole with a 10-mm diameter (78.5 mm^2^ in area), which closely matches the aortic valve area of the patient (approximately 72.0 mm^2^ on the CT image) and is usually classified as severe aortic stenosis^46^. To simulate the different angles of the aortic flows, a total of 9 different angles of the circular holes with the same diameter were fabricated (straight-, anterior-, posterior-, left-, and right-directional deflections at 15° and 30°) (Figure S1 in the Supplementary information).

The reconstructed 3D model of the thoracic aorta was fabricated using an acrylonitrile butadiene styrene-like material (VisiJetM3-X, 3D Systems, Rock Hill, SC) and a 3D printer (Projet 3510 SD, 3D Systems, Rock Hill, SC).

### Flow circuit system

Tap water was used as the working fluid to simulate aortic flow with a high Reynolds number (Re) by using relatively low viscosity. The density and the dynamic viscosity of the working fluid were 998.29 kg·m^−3^ and 1.0 × 10^−3^ kg·m^−1^·s^−1^, respectively. The working fluid was circulated through the flow circuit system at a constant flow rate using a centrifugal pump (Eheim Compact Plus 5000; Eheim, Deizisau, Germany). The flow rate was controlled using a flow valve and monitored using an electromagnetic flowmeter (VN20; Wintech Process, Seoul, South Korea). The flow rate was maintained at 8.3 ± 0.1 L/minute. This flow rate corresponds to Re around 5392. Re is expressed as Re = QD/νA, where Q is the flow rate, D is the diameter of the vessel, ν is the kinematic viscosity, and A is the cross-sectional area of the channel. Due to fluid-dynamic similarities, the fluid-dynamic features obtained by the present experiment are identical to features with the same Re. Therefore, the obtained flow is the same as the flow obtained using human blood viscosity (kinematic viscosity is approximately 3 × 10^−6^ m^2^·s^−1^), and the physiological systolic flow rate of Q is approximately ~24.9 L/minute^47^. Considering that the previous studies on aortic flows for normal and diseased patients reported that Re ranges around 5000 to 10 000^48^,^49^, Re of 5392 for the present study is within the physiological range.

### 4D PC-MRI flow imaging

4D PC-MRI measurements were performed using a clinical 3.0-T MRI scanner (MAGNETOM Skyra; Siemens, Munich, Germany). A gradient-echo sequence with 4-point velocity encoding (VENC) was used. VENC was set to 250 and 70 cm/s for the velocity and TKE measurements, respectively. TE and temporal resolution were 4.76 and 73.2 ms, respectively. The flip angle was 10°. The field of view was 178 mm × 272 mm × 120 mm with a 1-mm isotropic voxel size. Partial Fourier acquisition (a factor of 6/8 along the phase- and frequency-encoding directions) and integrated parallel acquisition technique with 32 reference lines for 2-fold acceleration along the phase-encoding direction were used. As a result, the total scanning time was approximately 8 minutes.

### Preprocessing and visualization

The obtained magnitude and phase data were loaded into MATLAB (MathWorks, Natick, MA). At first, second-order polynomial fitting was applied to the stationary part of the phase data to remove any eddy current-induced background phase offset^50^. Then, the region of interest (ROI)—i.e., the thoracic aorta—was segmented on the magnitude images, and the exterior region of the ROI was filtered out. Staring from the 3D velocity field, λ_ci_, WSS, impinging pressure, and TKE were calculated. The resultant data were exported and visualized using Ensight 10.1 (CEI, Apex, NC).

### Rotational velocity

The centerline skeleton of the thoracic aorta was obtained from the ROI mask images based on the accurate, fast-marching distance transformation. At each point of the skeleton, the axial directional vector was calculated from 2 consecutive skeleton points. Then, the 2D plane was generated using the axial directional vector as the normal vector, and the velocity data at the 2D plane were linearly interpolated. Then, the velocity data were decomposed into 3 axis: axial, radial, and tangential velocities. In the present study, the tangential velocity component was used as the rotational velocity of the flow around the centerline of the thoracic aorta.

### Vortex identification

Identification of the vortex flow from the velocity vector field was based on critical point analyses of the local velocity gradient tensor and its corresponding eigenvalues^51^,^52^,^53^,^54^. The local velocity gradient tensor of flow has 1 real eigenvalue (λ_r_) and a pair of complex conjugate eigenvalues (λ_cr_ ± iλ_ci_) when the discriminant of its characteristic equation is positive. λ_ci_^−1^ represents the period required for a fluid particle to rotate around the λ_cr_-axis^51^,^55^. Therefore, a non-zero λ_ci_ indicates the existence of a local swirling region, and the magnitude of λ_ci_ indicates the strength of the local rotation. Since λ_ci_ is Galilean-invariant, vortical motion of the flow can be quantified even when the flow is overlaid with the mean shearing flow, such as jet flow. Further information about the vortex identification method can be found in the study by Wu and Christensen^56^.

### WSS estimation

In this study, WSS estimation in the thoracic aorta from 4D PC-MRI was based on the method described by Ha *et al*.^57^. First, the 3D surface of the thoracic vessel was generated from the ROI mask images in order to estimate the wall locations (Figure S2 in the Supplementary Information). The 3D surface consisted of around 860,770 fine triangular surfaces. For each triangular surface, the central position of the surface and the normal directional vector were extracted. Then, velocity data along the normal direction were interpolated using spline interpolation, and WSS was estimated using the following equation:


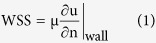


where n is the local wall-normal vector, u is the local velocity component, and μ is the dynamic viscosity of the fluid. To estimate the gradient of the velocity at the wall, the wall positions were set to zero-crossing points where the flow velocity was zero because of nonslip conditions.

### TKE and MKE estimation

TKE is estimated from the intravoxel standard deviation of the velocity (IVSD), which is estimated based on the turbulence-induced signal attenuation^58^,^59^. In the present study, the raw k-space data were exported from the MRI scanner, and an in-house MATLAB code was used to reconstruct the magnitude of the images using 4-point measurements (the reference and 3-directional measurements). The TKE per unit volume can be estimated from σ as follows:


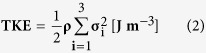


where ρ is the fluid density, and σ_i_ indicates the standard deviation of the velocity in the i-direction. Total TKE was obtained by integrating the TKE values over the thoracic aorta. In the preliminary study, the Siemens 4D PC-MRI sequence was found to eliminate TKE along the slice-direction because the sequence has nonsymmetric encoding around 2 axes. Therefore, we note that TKE in the present study could be lower in comparison with other studies that used other MR scanners^59^,^60^.

The mean kinetic energy of the flow can be obtained as follows^41^.


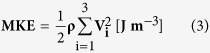


where V_i_ is the mean velocity component along the i-direction obtained by 4D PC-MRI. Therefore, the total kinetic energy can be obtained from the sum of TKE and MKE.

### Impinging pressure

Jet blood flow from the aortic valve tends to change its direction when impinging on the ascending aorta. According to the fluid-dynamic principle, the change in the fluid momentum at the vessel is balanced with the force exerted on the vessel according to the following equation:


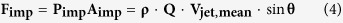


where F_imp_ is the force exerted on the vessel, P_imp_ is the impinging pressure, A_imp_ is the contact area of the impinging flow, V_jet,mean_ is the mean velocity of the jet blood flow at the impinging location, and θ is the impinging angle of the blood flow. In addition, the continuity of the fluid satisfies V_jet,mean_ = Q/A_jet_ and A_jet_ V_jet,peak_ ≈ A_goa_V_geo,peak_, where A_jet_ and V_jet,peak_ are the area and peak velocity of the impinging jet flow, and A_goa_ and V_geo,peak_ are the area and peak velocity at the exit of the aortic valve, respectively (see Figure S3 in the Supplementary Information). As a result, the increase in the local static pressure (P_imp_) by flow impingement can be estimated using the following equation:


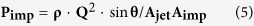


where the impinging area is estimated as A_imp_ = A_jet_∙sinθ. In the present study, considering that Q is fixed for the experiment, only θ and V_jet,peak_ were extracted from 4D PC-MRI data for P_imp_ estimation.

### Statistics

SPSS Statistics (version 17.0; IBM Corporation, NY) was used for the statistical analysis. Data were tested for a normal distribution using the Shapiro-Wilk test. Normally distributed data were analyzed using the student *t* test for 2-group comparisons. In this study, to account for twelve multiple tests for the dataset (as shown in Fig. 3B), we used the Bonferroni correction and considered significant only those for which *p* < 0.01/12 = 0.0008. Finally, it was described in the main text as *p* < 0.01, Bonferroni corrected.

## Additional Information

**How to cite this article**: Ha, H. *et al*. The influence of the aortic valve angle on the hemodynamic features of the thoracic aorta. *Sci. Rep.* **6**, 32316; doi: 10.1038/srep32316 (2016).

## Supplementary Material

Supplementary Information

## Figures and Tables

**Figure 1:**
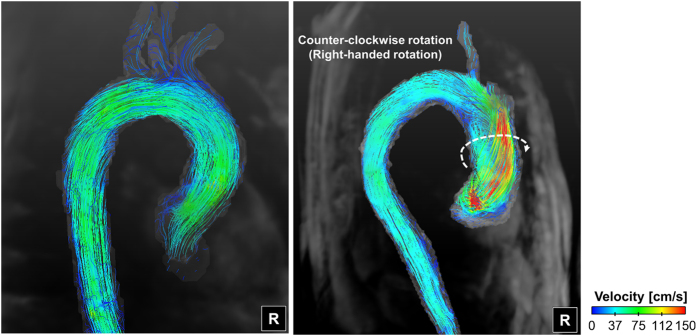
Representative helical flow pattern in the ascending aorta. R indicates the right direction.

**Table 1:** Experimental results.

Flow condition	Flow angle [degree]	0	15	15	15	15	30	30	30	30
	Flow direction	Straight	Anterior	Left	Posterior	Right	Anterior	Left	Posterior	Right
Rotational velocity	V_rot,AsAo_ [cm/s] (N = 826)	29.54 ± 14.73	21.36 ± 12.54*	28.70 ± 26.07	53.45 ± 27.28*	49.79 ± 24.54*	28.61 ± 17.07†	21.50 ± 19.46*,†	62.34 ± 31.32*,†	51.18 ± 29.69*
	V_rot,avg_ [cm/s] (N = 257,780)	5.56 ± 10.74	4.03 ± 8.28*	4.58 ± 10.67*	8.85 ± 17.36*	6.50 ± 15.67*,†	5.87 ± 11.83*,†	4.86 ± 10.26*,†	11.19 ± 19.63*,†	6.50 ± 15.67*,†
Impinging parameters	Impinging angle [degree] (N = 5)	46.13 ± 1.41	32.89 ± 2.35	11.96 ± 1.88	20.43 ± 0.76	35.93 ± 0.46	12.02 ± 2.17	38.63 ± 1.20	28.08 ± 0.90	21.18 ± 1.18
	V_jet,peak_ [cm/s] (N = 5)	94.02 ± 2.51	101.52 ± 3.98	149.82 ± 2.64	153.22 ± 4.00	124.28 ± 3.65	154.86 ± 3.48	154.86 ± 4.17	156.08 ± 2.26	151.42 ± 6.10
	V_goa,peak_ [cm/s] (N = 5)	220.00	248.40	243.50	237.50	238.30	231.20	235.30	235.30	234.30
	A_jet_ [cm^2^]	1.85 ± 0.05	1.80 ± 0.07	1.24 ± 0.02	1.21 ± 0.03	1.49 ± 0.04	1.27 ± 0.03	1.32 ± 0.04	1.20 ± 0.02	1.24 ± 0.05
	A_goa_ [cm^2^]					0.79				
	Q [L/min]					8.10				
	P_imp_ [mmHg]	4.86 ± 0.22	2.85 ± 0.48	0.61 ± 0.19	1.74 ± 0.18	4.00 ± 0.05	0.61 ± 0.21	5.18 ± 0.20	3.19 ± 0.24	1.83 ± 0.27
WSS	WSS 95% [Pa] (N = 4335)	0.52 ± 0.10	0.77 ± 0.10*	0.68 ± 0.22*	0.83 ± 0.13*	0.81 ± 0.11*	0.81 ± 0.09*,†	0.70 ± 0.23*,†	1.04 ± 0.30*,†	0.98 ± 0.09*,†
	WSS 5% [Pa] (N = 4335)	0.003 ± 0.006	0.004 ± 0.007*	0.005 ± 0.008*	0.004 ± 0.008*	0.003 ± 0.005*	−0.005 ± 0.006*,†	−0.004 ± 0.005*,†	0.006 ± 0.009*,†	0.006 ± 0.009*,†
	WSS_max_ [Pa]	0.86	1.07	1.48	1.20	1.08	1.18	1.52	2.06	1.45
Energy parameters	TKE_95%_ [mJ] (N = 12,889)	115.93 ± 18.24	100.20 ± 19.89*	105.02 ± 25.64*	111.76 ± 23.67*	115.77 ± 23.59	88.44 ± 22.54*,†	89.34 ± 17.70*,†	108.74 ± 25.79*,†	95.40 ± 24.60*,†
	TKE_max_ [J/m^3^]	228.28	265.08	244.30	217.02	199.98	258.25	233.44	229.47	254.80
	Total TKE [mJ]	7.47	5.64	5.81	6.83	7.20	4.95	5.42	6.64	5.59
	Total MKE [mJ]	14.72	16.05	16.39	22.73	19.08	16.21	14.80	26.38	19.18
	Total KE [mJ]	22.19	21.69	20.20	29.56	26.28	21.16	20.22	33.02	24.76

V_rot,AsAo_ = rotational velocity at the ascending aorta; V_rot,avg_ = averaged rotational velocity in the thoracic aorta; V_jet,peak_ = peak velocity of the jet flow at the impinging region; V_goa,peak_ = peak velocity of the jet flow at the exit of the aortic valve; A_jet_ = area of the jet at the impinging region; A_goa_ = area of the jet flow at the exit of the aortic valve; Q = flow rate; P_imp_ = estimated impinging pressure; N = number of elements.

^*^Statistically different (p < 0.01) in comparison with TKE in straight aortic valve flow.

^†^Statistically different (p < 0.01) in comparison with TKE in the same aortic flow direction, but at 15° of the aortic valve flow. Data indicates the mean ± SD.
