# Supplementary material for: The influence of the aortic valve angle on the hemodynamic features of the thoracic aorta
Source: Sci Rep. 2016 Aug 26;6:32316. doi: 10.1038/srep32316 (PMC4999809; doi:10.1038/srep32316)
Supplement: Supplementary Information [file srep32316-s1.pdf]

The influence of the aortic valve angle on the hemodynamic features of the thoracic aorta

Hojin Ha,[1] Guk Bae Kim,[2] Jihoon Kweon,[3] Sang Joon Lee,[1,4] Young-Hak Kim,[3] Namkug Kim,[5,6,†] and Dong Hyun Yang[6,†,*]

[1]POSTECH Biotech Center, Pohang University of Science and Technology, San 31, Hyoja-dong, Pohang 790-784, South Korea

[2]Asan Institute of Life Science, Asan Medical Center, University of Ulsan College of Medicine, 388-1 Poongnap-dong, Songpa-gu, Seoul 138-736, South Korea

[3]Department of Cardiology, University of Ulsan College of Medicine, Asan Medical Center, 388-1 Poongnap-dong, Songpa-gu, Seoul 138-736, South Korea

[4]Department of Mechanical Engineering, Pohang University of Science and Technology (POSTECH), San 31, Hyoja-dong, Pohang 790-784, South Korea

[5] Department of Convergence Medicine, University of Ulsan College of Medicine, Asan Medical Center, 388-1 Poongnap-dong, Songpa-gu, Seoul 138-736, South Korea

[6] Department of Radiology, University of Ulsan College of Medicine, Asan Medical Center, 388-1 Poongnap-dong, Songpa-gu, Seoul 138-736, South Korea

Criteria for submission: Original Articles

Submitted to *Scientific reports*

[*]**Corresponding author:** Prof. Dong Hyun Yang

Assistant Professor, Department of Radiology

University of Ulsan College of Medicine/Asan Medical Center

Tel.: +82-02-3010-6573; Email: donghyun.yang@gmail.com

†These authors contributed equally to this work.

Running title: Effect of the aortic flow angle on hemodynamic features

Number of words: 4009 words (not including abstract, Methods, References, and Figure Legends)

Number of figures and tables: 9 figures and 1 table

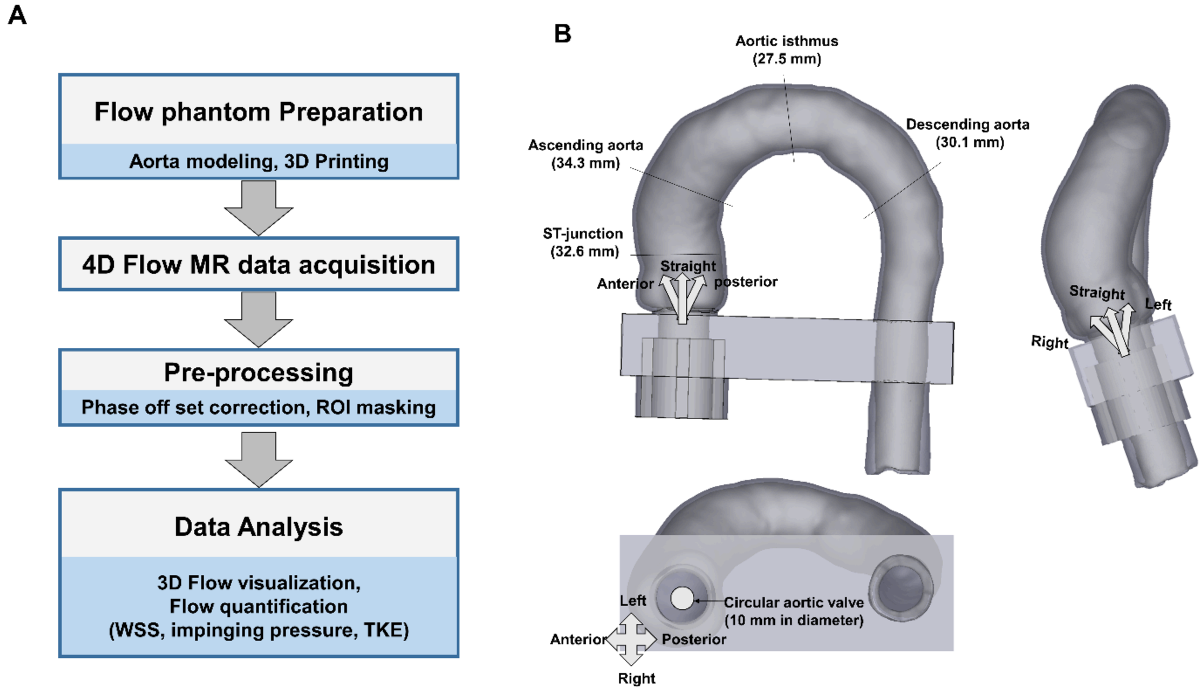

Figure S1. Schematics for the *in vitro* 4D PC-MRI experiments. (A) Experimental procedures and (B) *in vitro* aorta model with various aortic flow angles.

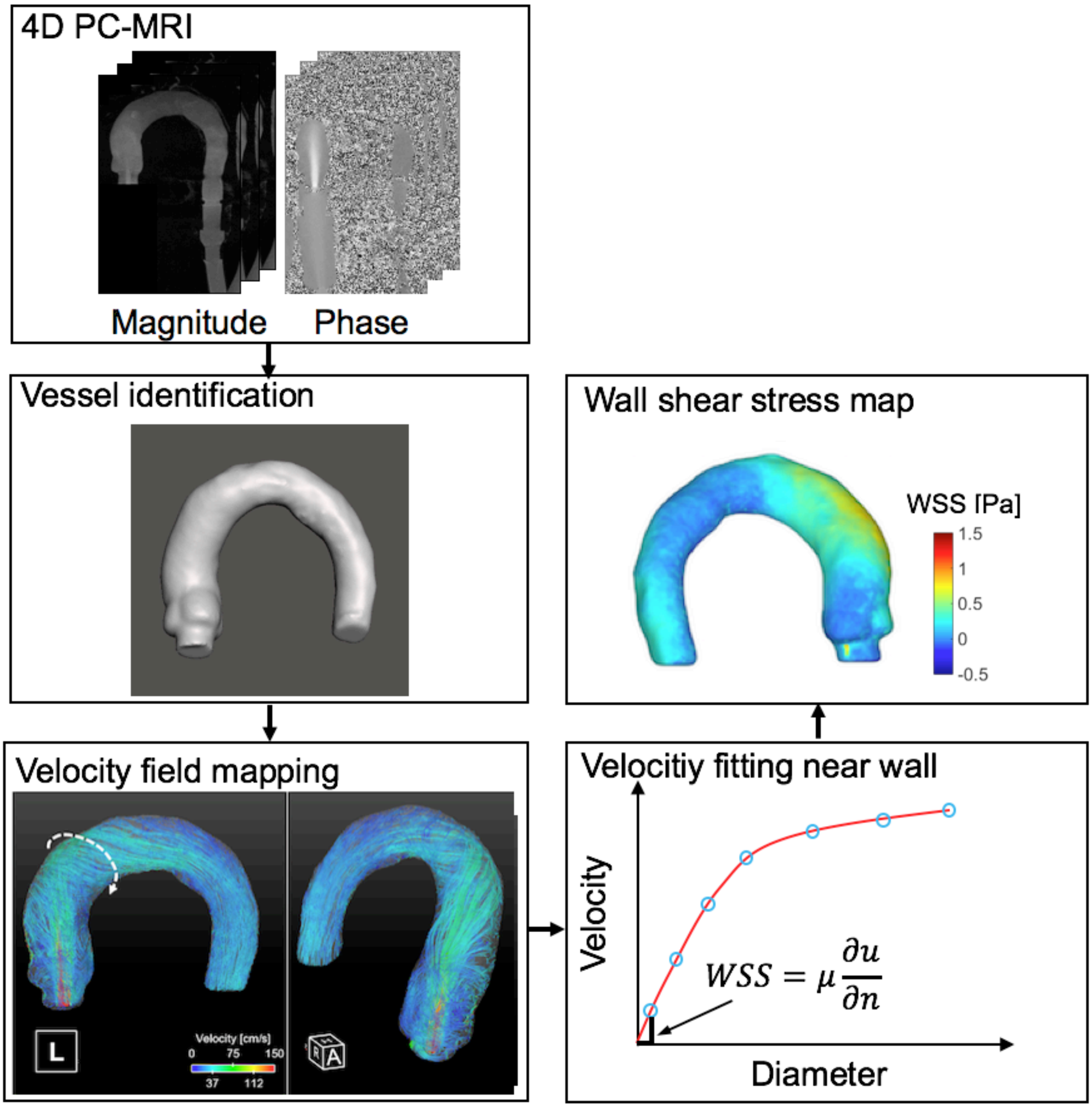

Figure S2. Schematic diagram for wall shear stress quantification.

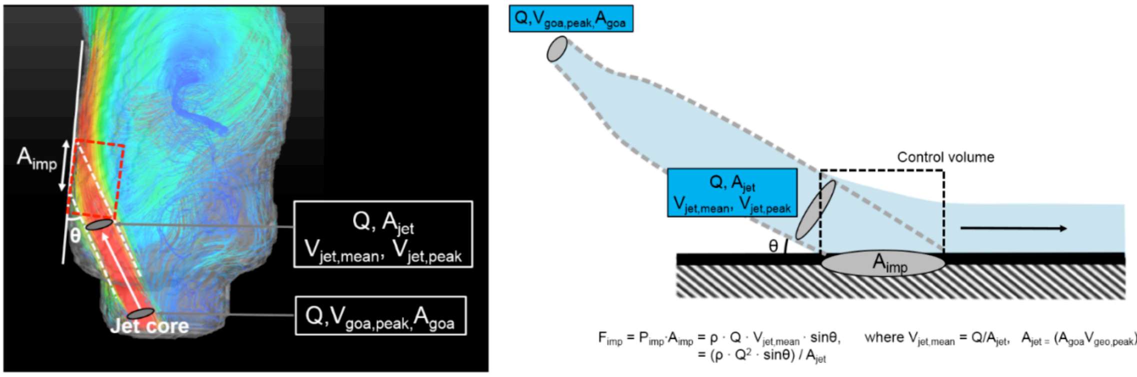

$$F_{imp} = P_{imp} \cdot A_{imp} = \rho \cdot Q \cdot V_{jet,mean} \cdot \sin\theta, \qquad \text{where } V_{jet,mean} = Q/A_{jet}, \quad A_{jet} = (A_{goa}V_{geo,peak})/V_{jet,peak}$$

$$= (\rho \cdot Q^2 \cdot \sin\theta) / A_{jet}$$

$$P_{imp} = (\rho \cdot Q^2 \cdot \sin\theta) / (A_{jet} \cdot A_{imp}), \qquad \text{where } A_{imp} = A_{jet}/\sin\theta$$

Figure S3. Schematic used to estimate impinging pressure.
